# Building collaborative prescribers: development and analysis of a novel simulation-based role exchange education programme between pharmacy and medical students

**DOI:** 10.1186/s41077-025-00399-3

**Published:** 2025-12-23

**Authors:** Niall O’Boyle, Peter Currie, Roisin O’Hare, Richard McCrory, Niall Leonard, Stephen Kirk

**Affiliations:** 1 South Eastern Health & Social Care Trust, Dundonald, Northern Ireland; 2 School of Pharmacy, Queen’s University Belfast, Belfast, Northern Ireland; 3 School of Pharmacy, University of Ulster, Coleraine, Northern Ireland

**Keywords:** Interprofessional learning, Medical students, Pharmacy students, Simulation, Role exchange

## Abstract

**Background:**

Undergraduate curricula across a number of healthcare professions have increased their focus on interprofessional education. From 2026 all pharmacy graduates in the UK will be independent prescribers, which will require them to develop skills of patient history taking and clinical assessment. Likewise, medical graduates will face increasingly complex prescribing challenges in an aging population with chronic illnesses, polypharmacy and personalised medicine. Developing these skills via collaborative practice is essential to meet the healthcare challenges of the future.

**Methods:**

We developed a novel interprofessional educational programme focused on medical admissions for final year medical and pharmacy students in a teaching hospital between January and March 2025. We utilised the concept of role exchange in simulation to foster development of mutual skills as well as enhanced professional identity formation and unique role recognition. We researched participant’s ability to work collaboratively, their skill development and their perceptions of IPL using a mixed methods approach. Pre and post course questionnaires using the Interprofessional Education Collaborative (IPEC) self-assessment tool were completed by participants in addition to questions on skill acquisition and free text questions regarding their experiences of the programme. Statistical analysis using independent t-testing was performed and a summary of narrative data was used to analyse the qualitative data.

**Results:**

The cohort size was 59 students (25 medical and 34 pharmacy). The pre-course response rate was 92% (*n* = 23) for medicine and 88% (*n* = 30) for pharmacy and the post course response rate was 88% (*n* = 22) for medicines and 91% (*n* = 31) for pharmacy. A total of 106 responses were analysed. A significant improvement was observed across: Interprofessional Values, Interprofessional Interactions and total score. Review of student comments identified several benefits and areas for future development. Students rated the course highly and 96% (*n* = 51) believed that they would be able to use the skills and knowledge gained in their future practice.

**Conclusions:**

Authentic simulated scenarios designed to align with professional practice, facilitated by role exchange, improves interprofessional collaboration and mutually develops skills in history taking and prescribing. This programme supports new standards for education in both medicine and pharmacy but delivery remains challenging due to its resource-intensive nature.

**Supplementary Information:**

The online version contains supplementary material available at 10.1186/s41077-025-00399-3.

## Background

Doctors and pharmacists bring complementary expertise to patient care, however uni-disciplinary modes of healthcare education delivery, and silos of preferred expertise, have often not prepared students for working in interprofessional clinical teams [1]. To address this difficulty, there is a need to provide students with interprofessional education (IPE) opportunities to learn with, from, and about other disciplines [2–4]. Whilst student IPE experiences have demonstrated evidence of better preparedness of both medical and pharmacy students for professional practice [5–7], some healthcare workers have criticised IPE initiatives for reinforcing imbalances in power between disciplines, rather than fostering collaborative practice [8]. As a result, positive learning outcomes are only possible when all student groups can participate and engage meaningfully in the educational opportunity. One concept in order to promote meaningful participation between disciplines is the use of role exchange, whereby students would adopt the role of a different professional within the IPE experience, in order to develop a greater insight into the experience and abilities of other team members [9]. As far as the authors are aware, the use of role exchange in simulation between medical and pharmacy students has not been described in the literature. We sought to develop an IPE programme incorporating role exchange to improve history taking and prescribing skills in both groups, and evaluate this process.

The disciplines of pharmacy and medicines have always worked alongside each other, and in many cases, responsibilities are now integrated between disciplines to include: gathering an accurate patient medical and medication history to facilitate a clinical diagnosis as well as development and prescribing of an appropriate clinical management plan [10–12]. Medication errors however remain a prominent safety concern, and can occur at many stages in a patient’s journey within the healthcare system. Medicine discrepancies on admission and on discharge from hospital have been identified as particularly high-risk moments for error [13], with nearly a third of patients having at least one medication error [14]. Rates of error have also been demonstrated to be higher in recently graduated medical prescribers [15]. It has however been demonstrated that pharmacists have a valuable role in safely and accurately prescribing inpatient medication at the point of admission to hospital [16]. A recent systematic review has therefore identified a specific need for IPE focused on safe prescribing to be integrated into undergraduate healthcare professional curricula [17].

Simulation is a well-established educational tool used within undergraduate healthcare profession education and is frequently applied for the purpose of IPE [18–20]. The premise of simulation is to enable learners to master a skill in a well-defined learning space before experiencing the “live” clinical environment. It allows students opportunities to develop their communication, consultation and clinical decision making skills in addition to receiving tailored feedback in a safe, supportive learning environment [21]. IPE opportunities, including between student groups in a simulated environment, may encourage mutual skill development between peers with different levels of knowledge and experience. Furthermore, adopting the role of a different professional in the safety of a simulation has been shown to promote student reflection on their own professional skills as well as understand the importance of interprofessional communication and collaboration [22]. The co-design of interprofessional simulations among multiple professions is however critical for fostering effective learning experiences for all participants [23]. Therefore, by involving all professional groups in the design of the simulation scenarios, and by targeting admission to hospital as a natural point where doctors and pharmacists work collaboratively in practice, this should ensure reflection of the skills and knowledge of each profession are explored and highlighted.

## Methods

### Aim

The primary aim of the study was to assess the impact of an IPE programme on student self-reported interprofessional collaboration when working together. Secondly, we aimed to ascertain student perceptions of the IPE programme as well as how well the IPE programme supported their clinical skills development.

### Design

The study used a mixed methods approach. Quantitative analysis was carried out on two instruments which both use a 1–5 Likert scale. Student interprofessional collaboration was analysed using a validated self-assessment tool both pre- and post IPE programme. The Interprofessional Educational Collaborative (IPEC) self-assessment tool (Version 3) (supplementary material) is a 16-item Likert Scale instrument scoring a participant’s purported competency related to collaborative practice from “Strongly Agree” to “Strongly Disagree”. This questionnaire has been validated repeatedly in large studies and covers two major themes – interprofessional interaction and interprofessional values [24].

Evaluation of student overall experience and skill development was also completed post IPE programme (supplementary material). The questions used to evaluate overall student experience and skill development were created independently by the researchers with a focus on student confidence, development of communication and consultation skills and benefits of the IPE programme for future experience. Quantitative analysis, to include descriptive statistics, were completed on the results of this evaluation.

Qualitative analysis occurred with two short answer questions included within the post IPE programme evaluation, which included the following questions: “What do you feel went well with the IPL activity between medical and pharmacy students?” and “How do you feel that the IPL activity between medical and pharmacy students could be improved in the future?”.

### Recruitment

Participants comprised final year medicine and pharmacy students selected using convenience sampling via allocation of undergraduate placements to a teaching hospital between January and March 2025. Participation in the IPE programme was voluntary for both medical and pharmacy students. Medical and pharmacy project leads, respectively, sent students an invitation email with a participant information sheet detailing the aims of the course, the study’s purpose and confidentiality measures. The email also contained a QR code link to an electronic consent form for agreement to participate in the study as well as an electronic Microsoft Forms pre-course questionnaire which students were asked to complete before attending the IPE programme.

### Description of educational activity

A full-day IPE programme focused on a safety-critical moment - medical history taking and reconciliation of medicines at initial hospital admission was designed. The first session comprised of four simulation-based case scenarios. Cases were based on the most common prescribing errors reported in local hospitals: anticoagulation, insulin, critical medications and adverse effects of polypharmacy. When students attended for the IPE programme they were pre-briefed by the medical and pharmacy project leads to include: details on the format of the day, the expectations of the simulation scenarios as well as the clinical sessions with patients on the wards and details of role swapping throughout the IPE programme. Interprofessional pairs, comprised of medical and pharmacy students, were then tasked with taking a medical history and performing medication reconciliation from faculty members playing the role of the simulated patient. Following this, they were required to formulate a clinical management plan for the patient’s medical admission. The simulated participant was given a script and the facilitator had various resources such as results of investigations to give to the students when appropriate. Faculty consisted of a pharmacist and a doctor to facilitate the scenarios and debrief the students using the PEARLS debriefing model [25]. The difficulty of the cases gradually increased throughout the first session. In addition to their learning focused on interprofessional collaboration, specific learning outcomes relating to medications safety were also identified in the debriefs.

Students adhered to their dedicated healthcare discipline for the first two scenarios, with the aim of allowing participants to observe their partners’ communication skillset and strategies to gain information. For the second two scenarios, participants exchanged roles, with the pharmacy student taking the medical history and the medical student performing the medication reconciliation. In the second session, the interprofessional pairs attended non-simulated hospital wards to interact with real patients. Faculty guided them to recently admitted patients, when possible, with whom they could conduct a medical history and complete a medicines reconciliation. Study participants again exchanged professional roles between patients interviewed. The programme concluded with a final facilitated student debrief by faculty, to include: review of learning outcomes and exploration of the impact of role swapping throughout the IPE programme. Following completion of the debrief, students were then invited to complete an electronic post-course questionnaire. A successful pilot took place in December 2024 with four students in which the simulations achieved the desired learning outcomes during facilitated debriefing and data collection of the electronic pre- and post-questionnaires was efficient. Following the successful pilot the IPE programme was then upscaled as described.

### Data capture and analysis

The collected numerical and free-text data was collected anonymously and stored on a secure university Sharepoint server.

We performed statistical analyses on the Likert scale data using SPSS software (version 24.0). The method of data collection placed specific constraints on statistical tests applied. The asynchronous mode of participants completing the questionnaire meant that pairing pre- and post course scores was not possible. Hence, we used independent t-testing to determine if there was a statistically significant change in the IPEC subscale and total score for each discipline following the intervention. Effect size was calculated using Cohen’s D.

Questions regarding student experience and skills development were also written in the positive, following the same format as the IPEC self-assessment tool (Version3), and were measured using a similar Likert scale. Data collected in relation to student experience and skills development was analysed using descriptive statistics.

Qualitative data from the written feedback was initially reviewed by two researchers from the study team (NOB and PC) independently. Analysis was conducted on the student free text narrative data in order to identify themes. Following the first independent review, the researchers met to agree emerging subthemes. The reviewers then met regularly to define and name developing themes. A summary of the narrative data as well as the identified themes have been included in the results.

### Reflexivity

The research team was led by a pharmacist (NOB) and a doctor (PC). They remained reflexive throughout the study by meeting regularly to discuss and work collaboratively to interpret the evaluations, and appreciated that an interprofessional approach as well as a recognition of their different backgrounds and perspectives could enhance the research. NOB is an educational pharmacist with an interest in simulation and PC is a specialty registrar in anaesthesia with an interest in simulation.

### Ethical approval

Ethical approval was obtained from the University Research Ethics Committee (Reference MHLS 24_175). In addition to informed consent from participants, letters of support were obtained from the respective schools of medicine and pharmacy from each university which permitted students to participate.

## Results

Three IPE sessions were delivered between January 2025 and March 2025. A total of 25 medical and 34 pharmacy students participated. Due to voluntary participation in the IPE programme there was an unplanned uneven split of pharmacy student and medical student interprofessional pairs – therefore in some circumstances two pharmacy students were paired with one medical student. The questionnaire response rate for medicine was 92% (*n* = 23) before and 88% (*n* = 22) after. For pharmacy it was 88% (*n* = 30) before and 91% (*n* = 31) after.

One medical student completed the pre-course questionnaire but was subsequently unable to attend. Their response was maintained within the data pool for analysis as it was anonymised, and deemed by the study team to reflect their baseline interprofessional competencies.

### IPEC scores

IPEC scores were analysed by subscales: Interprofessional Values and Interprofessional Interactions, and also by total score for both pharmacy and medicine cohorts (Tables 1 and 2).

**Table 1:** Analysis of pharmacy student IPEC scores

	Pre-Course Mean (SD)	Post-Course Mean (SD)	Student’ T (df)	*P* value	Cohen’s D	Effect Size Interpretation
Interprofessional Values	32.73 (5.36)	35.81 (3.53)	2.66 (59)	0.005	0.68	Medium
Interprofessional Interaction	30.4 (5.09)	35.06 (3.55)	4.16 (59)	< 0.001	1.07	Large
Total IPEC Score	63.13 (10.1)	70.87 (6.91)	3.5 (59)	< 0.001	0.9	Large

**Table 2:** Analysis of medical student IPEC scores

	Pre-Course Mean (SD)	Post-Course Mean (SD)	Student’ T (df)	*P* value	Cohen’s D	Effect Size Interpretation
Interprofessional Values	33.57 (2.45)	37.5 (4.22)	3.85 (43)	< 0.001	1.148	Large
Interprofessional Interaction	30.48 (3.01)	36.73 (4.11)	5.84 (43)	< 0.001	1.74	Large
Total IPEC Score	64.04 (4.73)	74.23 (8.21)	5.13 (43)	< 0.001	1.53	Large

A significant improvement in post-course IPEC scores was identified across both groups in the individual subscales and total score (marker for significance of *p* < 0.05). In the pharmacy group the increase in score for interprofessional values was found to be of moderate effect size by Cohen’s D (0.68) however for all other domains the effect size was large (1.07 and 0.9). For medicine the increase in score was of a large effect size for all domains. The large effect size in interprofessional interactions in both groups demonstrates the impact of the programme when they may not have had similar opportunities to interact with other professions in their undergraduate training prior to this. Whereas, although the effect size for interprofessional values was medium for pharmacy and large for medicine, it was proportionally smaller perhaps due to the teaching they receive on interprofessional values through their undergraduate training.

### Student experience and skills level

The mean score for their overall impression of the IPE programme was 4.75 out of 5 (with 1 being poor and 5 being excellent on the rating scale). The distribution across the student ratings included: 42 students rating the IPE programme as 5/5, 9 rating it as 4/5 and 2 rating it as 3/5. Among the 53 students who completed the questionnaire, 50 students (94%) agreed that the IPE simulation improved their self-reported confidence in completing the in-situ activities with real patients on the wards in the afternoon. In terms of exposure to IPE simulation within their undergraduate training, 92% (*n* = 49) of respondents would like more opportunities within their undergraduate degree training.

Overall, students reported that the IPE programme helped them gain insight into collaborative working practices. In total 96% (*n* = 51) of respondents reported that having a feedback conversation about the work of a multidisciplinary team member helped them to gain a different perspective on the roles of each other’s profession. Students were also able to understand the benefit of the IPE programme for their future training and working practices, with 96% (*n* = 51) of respondents reporting that they will be able to use the skills and knowledge gained in their future career.

### Analysis of narrative data

The responses to two questions “What do you feel went well?” and “What do you think could be improved?” were analysed by two independent researchers to identify themes. The first question on the benefits of the IPE programme identified three main themes as outline in Table 3 :interprofessional collaboration, professional identity as well as social and academic congruence.

**Table 3:** Themes identified from student reported benefits of the IPE programme and corresponding illustrative quotations

Themes	Subthemes	Illustrative quotation
Theme 1: Interprofessional collaboration	Team working	*Pharmacy student 27: “The medicine students were so supportive and really gave me an insight into good teamwork for optimising patient care.”*
	Collaboration	*Medical student 15: “I thought this was a valuable experience as a medical student… important to learn how to collaborate with pharmacists and learning how to work with other members of the multidisciplinary team. This was something that I had never done before and definitely thought it was worthwhile.”*
	Communication	*Pharmacy student 2: “Learning how to communicate with medical students and using both skill sets to reach the best decisions for patients.”*
	Multidisciplinary co-operation	*Medical student 20: “I think our collaboration felt very natural and I learned a lot about medicine reconciliation based on how the pharmacy student interacted in the SIMs and with the patients.”*
Theme 2: Professional identity	Role clarity	*Pharmacy student 4: “I realised that medical students aren’t as familiar with medications as we are and I can really see the role a pharmacist has.”*
	Knowledge and skills transfer	*Medical student 16: “Good respect for each other and how we can improve each others skills in history taking and prescribing.”*
	Occupational priorities	*Medical student 19: “I liked the role reversal too*,* it gave me an idea of the priorities of each profession and how to use these to benefit the patient.”*
Theme 3: Social and academic congruence	Social interaction	*Medical student 22: “This was a really great opportunity to come together with a another professional group that we will be working with as F1’s”*
	Peer teaching	*Pharmacy student 18: “Easier/less daunting to ask them questions than it is to ask doctors.”*
	Training alignment	*Pharmacy student 11: “It’s also nice the way they’ve been on placement too but are at the same stage where they aren’t responsible for anything yet but then the scenarios that we were doing was as if we were in the real world. Nice mix of pressure and also learning too.”*

The second question on future development of the programme identified two main themes as outlined in Table 4 :academic alignment and authentic experiences.

**Table 4:** Themes identified from student suggestions for the future developments of the IPE programme and corresponding illustrative quotations

Themes	Subthemes	Illustrative quotation
Theme 1: Academic alignme**nt**	Scheduling	*Pharmacy student 18: “Do it mid-week*,* not right before exams. Medical students seemed under pressure.”*
	Curriculum development	*Medical student 13: “This activity would have been beneficial for students in advance of the prescribing exam. It would have been beneficial for medical students to be critically assessed on history taking and examinations.”*
Theme 2: Authentic experience	Resource awareness	*Medical student 22: “It would be good to advise us on the different calculators apps that would be good to download before the day such as the BNF*,* EOLAS*,* Optimal”*
	Realism of simulation	*Pharmacy student 26: “Using epic to show how they would then document the history and how the medications would be reconciled.”*
		*Pharmacy student 23: “The use of an ECR would help pharmacy students.”*

## Discussion

Pharmacy and medical students reported significantly increased collaboration competency scores after the course. Whilst experiential learning placements currently afford students some exposure to the workings of other disciplines, the rise in mean competency score may suggest that uni-professional training remains the norm rather than the exception. Further work to investigate collaboration scores at several points throughout students’ clinical training in all disciplines might better distinguish this pattern. Determining if this increase is maintained or at what time it declines could then suggest how and when such IPE should take place.

Participants’ free-text responses were consistent with the quantitative findings. Student groups rated their experience of the IPE programme highly, described benefits of collaborating in simulated and authentic work settings, and desired more opportunities to learn prescribing skills together. The opportunity for role exchange during the activity contributed to an increase in competency scores. Notably, the effect size for interprofessional interaction saw the greatest increase, perhaps because the programme focused heavily on interactions between professions. Additionally, role exchange was intended to improve their knowledge of another profession which was explicitly assessed in the interaction subscale. The interprofessional values domain also saw a notable increase in both groups with a large effect size in medicine and medium effect size in pharmacy. This large increase in the respondent’s values following one IPE intervention warrants further longitudinal study with repeat assessments to see if this increase is maintained over time.

The themes identified in the qualitative analysis provided other insights that might aid students to be better prepared for practice. Notable examples were the lack of awareness some students had of potential supporting resources, such as dosing calculators, many of which are open-access. They also highlighted the role simulation can play in creating scenarios that not only provide opportunities to develop their interprofessional collaboration but also develop their clinical skills. Refining and enhancing the scenarios to make them more authentic to applied clinical practice was also emphasised in qualitative feedback.

Increasing complexity in patient management requires a degree of interchangeable skills for both doctors and pharmacists [12]. Collaboration aptly describes students learning to prescribe as a ‘whole-task’ with each discipline providing mutual support during the experience. Consequently, asymmetries of authority and responsibility became less prominent throughout this study, building upon previous research demonstrating the key role that pharmacists play within a collaborative healthcare team [26]. Positioned out of their identified healthcare role, into another healthcare role in a particular situation, students learn to integrate perspectives from an alternative viewpoint into their clinical reasoning and formation of clinical management strategies. Additionally, the results suggest that IPE activities using role exchange help students appreciate and develop their professional identity and foster co-operation with future colleagues in clinical teams with the purpose of providing safe, effective, and high-quality patient care. The focus on role exchange and authentic simulated scenarios in this study adds a novel dimension to the literature, primarily on the impact of the development of interprofessional collaboration, knowledge and skills in addition to improving medication safety. This provided students with opportunities to practice history-taking and medication reconciliation skills in a simulated setting, develop their understanding through collaborative endeavor with another discipline, and put their new skillset into practice in the clinical environment.

In addition to the benefits of professional role exchange, another theme identified was academic and social congruence where students learned from one another in a supportive environment. This concept could be applied to other interprofessional scenarios where there is a significant overlap in skillset such as physiotherapists and respiratory medicine or speech and language therapists and stroke medicine.

The development of these interprofessional simulation based education initiatives required significant investment into faculty in order to support and facilitate the simulation scenarios. Over the three dates the programme was delivered, twenty four faculty members facilitated the interprofessional simulations - with one facilitator from each profession in a group of four students. This highlights the resource intensive nature of our programme. However, the benefits of multiprofessional feedback within the debrief, and the opportunity for role exchange requiring multiple simulated scenarios, justified the relatively high proportion of facilitators to participants.

Whilst simulation based education is strongly imbedded within medicine teaching programmes, other professions often have less exposure to simulation training, which can make recruiting appropriate faculty challenging. Training is therefore required to ensure that all faculty involved deliver high-quality teaching. Whilst this barrier is initially difficult to overcome, once trained and able to competently deliver simulation based education, we believe these professionals will provide excellent value to their organisation and improve access to the benefits of simulation both for their profession and those they engage in IPE with.

### Limitations

Despite the multi-faceted approach of this study, there are a number of limitations. Data collection was completely anonymised - meaning we were unable to pair the data. While it was possible to analyse the data via independent T testing, it was not possible to perform a paired T test, which may have increased statistical power by controlling for individual variability. Another limitation was the relatively fragmentary nature of the qualitative data, rather than in-depth analysis of interviews or focus groups.

The timing of the programme was tailored to students being present on hospital sites. However, the programme was competing with other assessments taking place - including the prescribing safety assessment and medical finals, which may be responsible for some of the pharmacy students finding their medical colleagues to be distracted. This is a limitation that could be mitigated in the future with improved scheduling. In addition to timing, running the programme in more hospital sites to increase the number of students, thereby increasing the sample size, could improve the validity of the results were further research to be undertaken.

This research was voluntary, and as such is open to self-selection bias. It is possible students who are more enthusiastic and engaged in developing their skills were more likely to volunteer to take part. As with all studies, response bias is also a possible limitation, with students giving what they see as desirable answers knowing they will be asked questions before and after.

Feedback from the faculty on the programme would have been useful to gain their perceptions on its strengths and future development. It could have also provided an insight into the experience and confidence of both professions in delivering simulation-based education. Formal research and analysis, however, would have required additional ethical approval from their employing organisation - which was not sought, but could be explored in future studies.

### Future development

Hospital discharge, like hospital admission, is another high risk point in the patient journey, and it is essential that accurate and robust communication strategies exist between primary and secondary care in order to minimize the potential for medication related error. Previous research suggests that as little as 26.9% of hospital discharge summaries identify reasons for medication changes [27]. Therefore, it is important to consider other high risk areas of interprofessional activity such as hospital discharges within future work within the undergraduate curriculum. In Northern Ireland, a new electronic health record system has also been rolled out between late-2023 and mid-2025 across the province. Developing educational opportunities that truly prepares students for practice will necessitate integration of this system within future healthcare simulation.

The level of realism within healthcare simulations is a critical factor to influence participant engagement and potential for learning [28]. Within Northern Ireland, the use of patient Electronic Care Records is often used to verify patient prescribed medication. Further development of simulation scenarios to include this resource would be worthwhile in order to enhance simulation realism for participants. The use of shared teaching materials for both medical and pharmacy students, such as dosing calculators and national guideline Apps, could also be considered in the future so that students are familiar with these resources prior to participation in the IPE programme.

## Conclusions

There is evidence that simulation will be a core component in undergraduate IPE efforts to address learning needs across both disciplines as it provides the opportunity to create scenarios with collaborative learning outcomes in a controlled environment. Students exchanged roles throughout the designed IPE programme, which gave them a greater insight into the roles and responsibilities of their colleagues. The qualitative data highlighted how simulation can effectively be used to develop rapport and understanding within interprofessional student cohorts before in-situ experiential learning. This mixed methods study has identified: a significant improvement in interprofessional collaboration through participation in the IPE programme, several benefits of IPE for undergraduate healthcare students and areas for future development.

## Supplementary Information

Supplementary Material 1.

Supplementary Material 2.

## Data Availability

The authors are willing to provide research data on reasonable request.

## Abbreviations

IPE: Interprofessional Education
IPEC: Interprofessional Education Collaborative
