# Supplementary figures and images for: Building collaborative prescribers: development and analysis of a novel simulation-based role exchange education programme between pharmacy and medical students
Source: Adv Simul (Lond). 2025 Dec 23;11:5. doi: 10.1186/s41077-025-00399-3 (PMC12837176; doi:10.1186/s41077-025-00399-3)

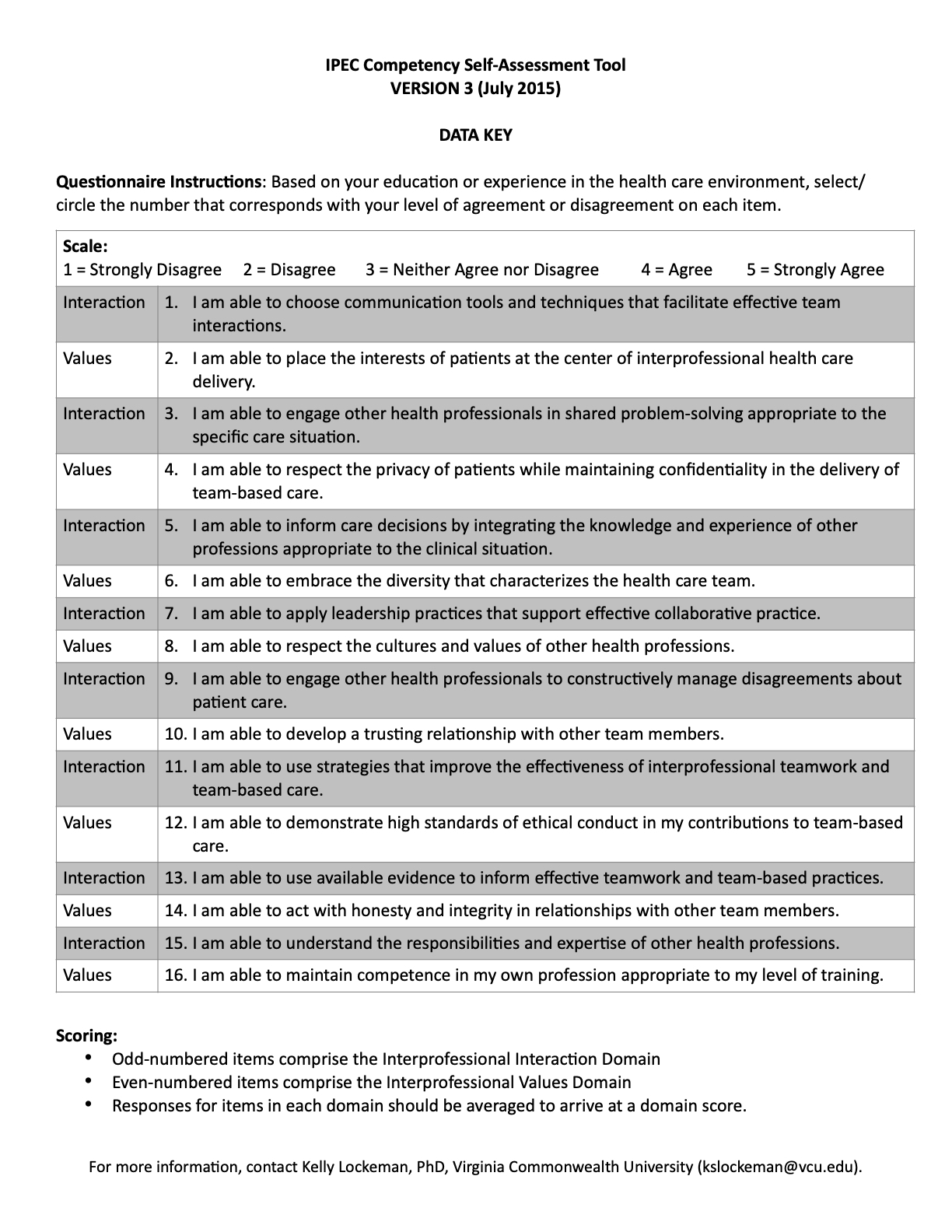


**Figure 1.** IPEC self-assessment tool (Version 3)

Supplement: Supplementary file 1 — Supplementary Material 1. [file 41077_2025_399_MOESM1_ESM.docx]
