# Supplementary material for: Building collaborative prescribers: development and analysis of a novel simulation-based role exchange education programme between pharmacy and medical students
Source: Adv Simul (Lond). 2025 Dec 23;11:5. doi: 10.1186/s41077-025-00399-3 (PMC12837176; doi:10.1186/s41077-025-00399-3)
Supplement: Supplementary file 2 [file 41077_2025_399_MOESM2_ESM.docx]

Table 5.  Post IPE Programme evaluation of student overall experience and skill development

| Overall rating |  |  |  |  |  |
| --- | --- | --- | --- | --- | --- |
| Please rate your overall impression of the IPL experience between medical and pharmacy students |  |  |  |  |  |
| IPL skills development | Strongly disagree | Disagree | Neither agree or disagree | Agree | Strongly agree |
| The IPL SIM improved my confidence in completing the in-situ IPL activities with real patients on the hospital wards |  |  |  |  |  |
| I would like more IPL SIM opportunities within my undergraduate degree training |  |  |  |  |  |
| The IPL activities on patient medical history taking and medicines reconciliation were relevant to my profession |  |  |  |  |  |
| The IPL activity on medical history taking improved my patient consultation skills |  |  |  |  |  |
| The IPL activity on medicines reconciliation improved my understanding of a patient’s therapeutic management when in hospital |  |  |  |  |  |
| Having a feedback conversation about the work of a multi-professional team member helped me to gain a different perspective on the roles of different professions |  |  |  |  |  |
| The IPL teaching and learning experience helped me to develop my communication skills as a multi-professional team member |  |  |  |  |  |
| I feel confident about working with other healthcare professionals |  |  |  |  |  |
| I will be able to use the skills and knowledge gained in my future experiential placements |  |  |  |  |  |
| I will be able to use the skills and knowledge gained in my future career as a registered healthcare professional |  |  |  |  |  |
